# Extending health systems resilience into communities: a qualitative study with community-based actors providing health services during the COVID-19 pandemic in the Philippines

**DOI:** 10.1186/s12913-022-08734-4

**Published:** 2022-11-21

**Authors:** Victoria Haldane, Warren Dodd, Amy Kipp, Hannah Ferrolino, Kendall Wilson, Danilo Servano, Lincoln L. Lau, Xiaolin Wei

**Affiliations:** 1 Dalla Lana School of Public Health, University of Toronto, 155 College St, M5T 3M7 Toronto, ON Canada; 2 School of Public Health Sciences, University of Waterloo, 200 University Ave West, N2L 3G1 Waterloo, ON Canada; 3 International Care Ministries, Unit 1701, 17th Floor, West Tower, Philippine Stock Exchange Centre, Exchange Road, Metro Manila, 1605 Pasig City, Philippines

**Keywords:** Non-governmental organizations, Community-based health services, Infection prevention and control, Public health emergencies, Health workforce, COVID-19 pandemic, Southeast Asia

## Abstract

**Background:**

Amidst ongoing calls for increased health systems resilience, gaps remain in our understanding of how health systems can reach further into communities to ensure resilient service delivery. Indeed, public health emergencies caused by infectious hazards reveal both the value and vulnerability of the workforce delivering health services in communities. This study explores ways in which a non-governmental organization (NGO) in the Philippines protected their frontline workforce during the first year of the COVID-19 pandemic.

**Methods:**

Guided by a qualitative descriptive approach, 34 in-depth interviews were conducted with community-based health actors employed by the NGO between June 2020 and February 2021. Data analysis was guided by an iterative deductive and inductive approach.

**Results:**

We identified four key activities that enabled the NGO and their staff to provide health and social services in communities in a safe and consistent manner as part of the organization’s pandemic response. These include (1) ensuring adequate personal protective equipment (PPE) and hygiene supplies; (2) providing contextualized and role-specific infection prevention and control (IPC) training; (3) ensuring access to testing for all staff; and (4) providing support during quarantine or isolation.

**Conclusion:**

Learning from the implementation of these activities offers a way forward toward health emergency preparedness and response that is crucially needed for NGOs to safely leverage their workforce during pandemics. Further, we describe how community-based health actors employed by NGOs can contribute to broader health systems resilience in the context of health emergency preparedness and response.

**Supplementary Information:**

The online version contains supplementary material available at 10.1186/s12913-022-08734-4.

## Background

The COVID-19 pandemic has challenged the resilience of health systems globally. Health systems resilience has historically been conceptualized as the ability of health systems to respond to and absorb shocks, crises, and emergencies, without compromising essential health services in communities [1, 2]. However, there is a broader literature which emphasises that resilient systems not only cope but also learn from and adapt to changing conditions [1, 3, 4]. The unprecedented duration and intensity of the COVID-19 pandemic has led to calls for strengthening health systems resilience as a key element of pandemic preparedness and response [5–7]. As part of this strengthening, many have pointed to the crucial role of community health workers in extending the reach of health services and systems further into communities before and during emergencies [8].

Community health workers, by which we mean lay members of the community who work either for pay or as volunteers in association with the local health care system, are but one part of a health workforce ensuring health and well-being in communities, particularly in low-and-middle-income countries (LMICs) [9]. In many LMICs, non-governmental organizations (NGOs) provide health education, promotion, referrals, and services [10, 11]. During outbreaks and pandemics, community-based health actors employed by NGOs are activated and use their status as trusted messengers to deliver public health messages in communities, link individuals to care in the health system, and ensure ongoing access to social and economic supports [12, 13]. As such, these community-based health actors are crucial agents of health systems resilience in communities, able to reach and work within communities to respond to shocks and ensure ongoing service provision [14].

While playing an important role in community health and well-being at all times, emergencies reveal both the value and vulnerability of the health workforce in communities [11, 13, 15, 16]. COVID-19 has demonstrated global challenges in keeping health workers safe, with estimates of up to 180,000 health worker deaths by the end of 2021 [17]. These estimates may be conservative figures if we broaden the scope to include community health workers and other community-based health actors. Given pervasive vaccine inequity, the NGO workforce in many LMICs must continue to rely largely on public health measures such as masking, hygiene, and ventilation for protection [18, 19]. With gaps in national and local government capacity in some LMICs, the NGO workforce has been mobilized during the pandemic to meet needs associated with disrupted access to routine health services, deepening poverty, and food insecurity [11]. These workers must be supported to carry out their ongoing work, while being safely equipped to face the demands of a pandemic.

Health systems resilience will continue to be challenged without greater consideration of how to protect this workforce from infectious hazards, particularly during prolonged emergencies such as the COVID-19 pandemic. This study explores ways in which an NGO in an LMIC protected their frontline workforce during the first year of the COVID-19 pandemic. We synthesize key lessons to inform emergency preparedness and response that is crucially needed for NGOs to safely leverage their workforce during the pandemic.

## Methods

### Study setting: International Care Ministries

**Fig. 1:**
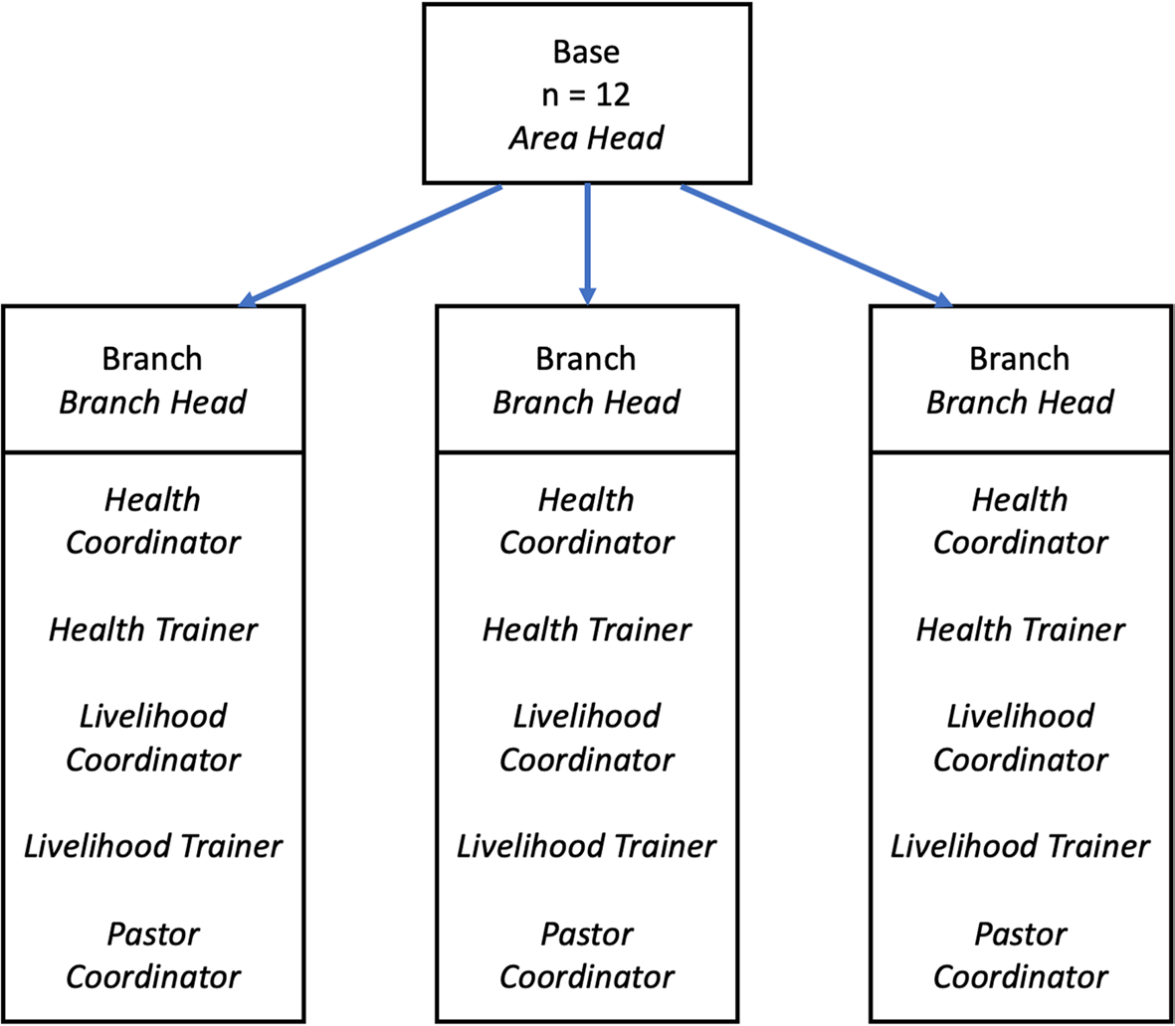
Simplified service delivery organizational chart for International Care Ministries

International Care Ministries (ICM) is an NGO based in the Philippines that operates programs focused on poverty alleviation with individuals and households experiencing ultra-poverty (defined by ICM as those living on less than 0.50 USD or 22 Philippine pesos per person per day) [20, 21]. ICM's core program is called Transform, which is a 15-week program that provides weekly health and livelihood education sessions for approximately 30 individuals per community. Leveraging its organizational strengths in community engagement and outreach, as well as collaboration with local government units (LGUs) and national government departments (e.g., Department of Health (DOH)), ICM delivers its programming through 12 bases across the country. Each base is led by an area head who supports three branches staffed by a branch head, health coordinators and trainers (focused on health education, promotion, referrals, and services), livelihood coordinators and trainers (focused on addressing socioeconomic needs), and pastor coordinators (focused on identifying potential partner communities) who provide services in communities (Fig. 1). Although each role has distinct responsibilities, all staff share the responsibility of supporting and enhancing the health and well-being of individuals and households participating in the Transform program (i.e., all staff can be considered community-based health actors). On average, 15 distinct Transform programs operate from each branch at any given time.

### Study setting: COVID-19 in the communities where International Care Ministries (ICM) works

Following the first reported case on 7 March 2020, COVID-19 rapidly spread across ICM programming areas [22, 23]. During this initial phase of the pandemic, decision-makers at all levels in the Philippines implemented a variety of public health and social measures. These included ‘blanket policies’ enacted at the national level, to more ‘granular policies’ and real-time interventions implemented at the local level through LGUs [24]. Amongst these were strict border controls, including across internal borders, that restricted population mobility between cities and regions. Known as community quarantine (CQ), these measures suspended ground, air and sea travel with compliance enforced by military and police personnel at checkpoints [25, 26]. As case numbers rose, Enhanced Community Quarantine (ECQ) was enforced, wherein residents were not allowed to leave their houses except to access essential goods and services [24]. These restrictions disproportionately affected the most vulnerable populations, who comprised participants in ICM's programs prior to the COVID-19 pandemic. However, with restrictions limiting people’s ability to work, obtain food, and seek health care and other social services, demand for aid grew in communities where ICM works [27].

The COVID-19 pandemic required ICM to safely adapt its usual programming to comply with public health mandates and implement COVID-19 related services, while simultaneously providing an increasingly important connection or pathway to the formal health care and social service systems for the communities they serve. To do so required significant efforts to ensure compliance with travel requirements needed to cross checkpoints into communities, while protecting the health and well-being of ICM staff, including acquiring and distributing personal protective equipment (PPE), ensuring staff received adequate infection prevention and control (IPC) training, and implementing COVID-19 testing for staff. Based on early guidance from China and then following guidance from the World Health Organization (WHO) and local health authorities, staff were given surgical masks and plastic face shields to be worn at work and when in communities. Masks were largely procured from local manufacturers. In addition, through a collaborative and iterative process, contextualized and role-specific IPC training materials for ICM staff were developed in early 2020 [28]. Training sessions were held in local languages and included demonstrations or ‘role play’ of key actions including donning and doffing PPE, hand washing, and ensuring public health guidance was followed in communities.

In late August 2020, the DOH mandated that frontline priority workers (“…those who… have high interaction with and exposure to the public”) needed a reverse transcription polymerase chain reaction (RT-PCR) test and highly encouraged employers “to regularly send their employees for testing once every quarter, at no cost to the employees” [29]. ICM staff were notified about these protocols through a formal presentation. Beginning in September 2020, all staff completed RT-PCR testing every three months wholly subsidized by ICM. As per protocols, all staff were required to be tested at an accredited/licensed laboratory and ICM bases were required to send a completed Workplace Accident/Illness Report on COVID-19 to the Department of Labour and Employment (DOLE) and the Department of Health (DOH) each month. By October 2020, COVID-19 case numbers had dropped across the areas where ICM operates, and an adapted Transform program was able to resume, which included smaller gathering sizes and house-to-house visits to monitor specific health outcomes (i.e., child malnutrition, maternal health, tuberculosis). The IPC training and periodic testing put in place by ICM enabled the approximately 400 ICM staff who come into direct contact with community members (e.g., trainers, coordinators, etc.) to safely resume field work activities in communities.

### Qualitative methods, participants, recruitment, and analysis

To explore the ways in which ICM protected their frontline workforce during the first year of the pandemic our analysis adopted a qualitative descriptive approach. This approach has been applied in similar cross-cultural research as it focuses on rich and precise descriptions of participants’ experiences with limited but thoughtful interpretation [30].

Participants were invited to the study by email or by phone call with a Tagalog speaking member of the research team. Ethical approval for this study was obtained from Office of Research Ethics at the University of Toronto, Canada (Ref: 20291), the University of Waterloo, Canada (Ref: 42061) and the Independent Ethics Committee at De La Salle Medical and Health Sciences Institute, Philippines (Ref: 2020-14-06-A). The information and consent form were available in English and Tagalog, and permission to be audio-recorded and quoted anonymously in research outputs was collected.

Interviews were conducted between June 2020 and February 2021 over videoconference by trained researchers from the study team, using a pre-designed interview guide covering access to care related constructs (Additional File 1). Interviews were semi-structured to allow for exploration of unanticipated themes or emergent areas. After obtaining informed consent, the interviews were recorded. Depending on participant fluency and comfort, interviews were conducted in English, Tagalog, Hiligaynon, Cebuano, or a mix of languages.

Five ICM bases were selected for geographic representation and COVID-19 related epidemiological characteristics, as well as established ties with the research team. We recruited participants from one branch within each base and each interview was conducted by at least two team members. Due to a strong organizational culture surrounding monitoring, evaluation, and research, interviewees were familiar with research processes and how research can contribute to collective learning and program improvements. Further, additional effort was made to build rapport with interviewees due to the remote nature of data collection.

To enhance validity and analytical rigour, four research team members (inclusive of Canadian researchers and ICM staff) met regularly during data collection to engage in peer debriefing and discuss observations across interviews [31]. Interviews not conducted in English were translated and transcribed in full, checked for accuracy by a multilingual research team member, and shared with the research team. After data familiarization, two research team members undertook an iterative process of coding the data that was both deductive (based on our understanding of the literature) and inductive (through close reading of the transcripts) to establish themes [32]. Codes were then discussed and grouped into thematic areas, from which themes were established. The two research team members discussed the themes to ensure they represented their shared understanding of the data and codes. We organized and coded data using QSR NVivo 12 software.

## Results

A total of 34 interviews were conducted with ICM staff providing frontline services during the COVID-19 pandemic, as well as managers and administrators at selected bases and branches (Table 1).

**Table 1:** Characteristics of International Care Ministries’ staff who participated in qualitative study (*n* = 34)

Participant Characteristics	Total (*n* = 34)
**LOCATION**	
Bacolod	11
Cebu	5
General Santos	6
Illoilo	6
Koronadal	6
**ROLE**	
Area head	4
Branch head	5
Health coordinator	8
Health trainer	9
Livelihood coordinator	2
Livelihood trainer	3
Pastor coordinator	3

Based on our analysis, we identified four key activities that enabled ICM and their staff to provide health and social services in communities in a safe and consistent manner as part of the organization’s pandemic response. Table 2 provides a summary of these areas with illustrative quotations.

**Table 2:** Illustrative quotations of key activities implemented by International Care Ministries during the COVID-19 pandemic

Activity	Illustrative quotation
Ensuring adequate personal protective equipment (PPE) and hygiene supplies	“So, we have supply coming from Manila, from the national office, we have some donation but you know the challenge is when it takes time to arrive in the province but as you know, with this face mask, alcohol, the face shield you know other companies and other providers they already contacted us if you have the supply, if you need this so we can give you a good price on that. So, before we implemented the program, we made it sure that we have the supply,” *PS008_Branch Head.*
Providing contextualized and role-specific infection prevention and control (IPC) training	“Our training it’s good for us because we know that we cannot see our enemy, so we have, um knowledge about the protocol about COVID-19… you can spread the contamination of the virus if ever your participants have flu or a cough, so much better that there is a social distancing so that the contamination of the virus cannot go directly to you,” *PS030_Health Coordinator*
Ensuring access to testing for all staff	“At first, I had fear because as what could you see in the tv, social media, news, all the negative things about swab testing especially when the test schedule is getting closer but when I tried it, it’s not really that painful, that other say that it will bleed, it didn’t and it was only mild. I really appreciate ICM for the welfare of the staff that when we go to the community, I need to be sure that I cannot infect them and my family. ICM also provides the payment that you will not spend any single peso from your wallet. And that is for the safety purposes both to the community and me,” *PS017_Livelihood coordinator*
Providing support during quarantine or isolation	“After I was tested positive, ICM provided groceries to my family for 2 weeks… Our area head said, I will just ask them if I need counselling. They can provide that [also],” *PS014_Health trainer.*

### Ensuring adequate personal protective equipment (PPE) and hygiene supplies

Safely providing services in communities during an outbreak requires ongoing access to PPE and hygiene supplies. All staff reported that being provided with PPE enabled them to safely carry out their duties and ensured that those participating in ICM programs were adequately protected. As one Area Head described, “We have face masks, we bring extra face masks, so we also have alcohol on the table…so they already observe the protocol” (*PS008_Area Head)*. Another Branch Head described how having PPE and protocols in place helped to ease staff hesitations, explaining “There’s fear inside but eventually as we talk, as we explain…we let them know that we cannot do this [community visits] if you are not prepared. You really need to prepare physically and then we have masks and things like that” (*PS012_Branch Head).*

Trainers echoed this sentiment, reporting that ongoing access to adequate PPE helped to overcome fear. For example, one Health Trainer explained, “We’re no longer afraid or worried about our safety plus we are also equipped with safety gears and the things that we need when we go to the community. So far, my worries for my own safety have lessened” (*PS004_Health Trainer)*.

While PPE was embraced by ICM staff at all levels, staff working in communities had to navigate the social perceptions of PPE use during the early stages of the pandemic. Reflecting on perceptions and understandings of PPE among participants in ICM’s programs, one Livelihood Trainer described how community members reacted to seeing someone in PPE:

“The positive [of wearing PPE] is when you reach the boundaries, you will be respected because you are wearing a PPE. When reaching the community, other people will think that there’s another COVID-positive in their area to be fetched [by Department of Health], so they’re afraid. I just explained to them that I am not from DOH. I am from ICM. I use PPE to protect you, to protect everybody who will interact with me. They later on understand” (*PS015_Livelihood trainer).*

All leaders described their role in coordinating PPE supplies, as well as in ensuring that PPE was worn, and protocols followed. Branch and Area Heads emphasized the importance of the ICM national office supplying PPE to the bases. However, despite these efforts to ensure universal access to PPE across branches, the reality of disrupted supply chains prompted some branches to take a proactive approach. One Branch Head, from a remote branch, described how leadership staff proactively worked with local suppliers early in the pandemic to maintain adequate stocks of PPE while waiting on supplies from the national office to arrive. In other branches there were reports of delays or limited PPE supplies causing staff to supplement with their own masks.

### Providing contextualized and role-specific infection prevention and control (IPC) training

Beyond providing PPE and supplies, IPC guidelines and complementary role- and context-specific training are essential to protecting community-based actors who provide health education, promotion, referrals, and services. In the early stages of the COVID-19 pandemic, ICM, in collaboration with external academic partners, developed and implemented role-specific guidelines for all their staff, with particular attention to the importance of IPC guidelines for staff engaged in health related outreach work in resource-constrained settings [28]. Staff were introduced to the guidelines via a series of training videos that presented step-by-step instructions of current IPC protocols.

When asked about the quality of training received regarding the IPC guidelines, all staff indicated that they felt the training contributed to enhanced confidence to conduct their work. For example, a Health Coordinator explained that “the training we have is very effective,” in relation to informing her work and the work of her colleagues. Of note, a different Health Coordinator spoke about the importance of continuing education and frequent training so that staff could be updated on new protocols and reminded to maintain high IPC standards across community settings.

Interviewees frequently discussed the relationship between ICM’s IPC guidelines and other external protocols that were imposed by LGUs. Reflecting on early experiences during the pandemic, interviewees from a branch associated with the Bacolod base focused on challenges associated with moving across cities and municipalities in the province of Negros Occidental to reach and support former Transform program participants. In particular, interviewees from this branch described how government officials required that they show weekly medical certificates at government-run checkpoints prior to entering a new region in the province. Moreover, interviewees emphasized how frequent and open communication with government officials played an important role in restarting their work following the lockdowns. Part of this communication with government officials included sharing how IPC guidelines would be followed when working in resource-constrained settings. Indeed, some interviewees connected their IPC training to these additional protocols and saw the IPC training as preparation for navigating external protocols to conduct their work. Overall, this commitment to engagement and trust building with local governments was a pillar of ICM’s approach prior to the pandemic, and provided a critical foundation for ongoing community-based work that adhered to external protocols amid the uncertainty of the pandemic.

Another important theme raised among most interviewees was how IPC training informed their work in communities, including the information provided to Transform participants and other community members. In all their community work, interviewees explained how they attempted to uphold IPC guidelines; however, variations were noted in terms of adherence to the guidelines across communities. For example, a Health Trainer explained:

“I always remind [program participants] that we should observe social distancing and wearing a face mask, but often times they are complaining “we cannot breathe” when they wear their face masks and sometimes, I keep telling them that they really have to observe the protocol and some they told me “ma’am it’s already normal, it’s only a flu.“ But for us as ICM staff we’re really obliged to go with the protocols because it’s our guidelines that we should have observed it, so for me as a health trainer, I see to it that I should be the first to model them” (*PS031_Health Trainer).*

This Health Trainer acknowledged the value in modelling the IPC guidelines with program participants as one approach to encourage adoption and adherence to these guidelines in the places where she worked.

A lack of adherence to IPC guidelines in communities was not always a product of misinformation or limited awareness surrounding disease severity. A different Health Trainer acknowledged how poverty intersected with the ability to adhere to IPC guidelines, “…in the community…the poor…it is hard for them…to buy face masks, face shield[s]” (*PS032_Health trainer).* In this example, the Health Trainer shared that she encouraged program participants to maintain physical distance as this was a cost-free approach to limiting virus transmission.

Interviewees also acknowledged how they were one of several actors providing COVID-19-related education and support within resource-constrained settings. Volunteer faith leaders and *barangay* health workers (BHWs; i.e., community health workers) were frequently mentioned as actors providing complementary supports to individuals experiencing poverty in the context of the pandemic. However, interviewees often saw their role as distinct from these other actors. For example, one Health Coordinator described how her role was focused on education and referring symptomatic individuals for further care at local health facilities. She explained:

“With the work of the barangay health worker and with the work of the health coordinator is different. So as a health coordinator, because, in the ICM, in our work ...we just educate them, channel them…We have the partnership with the ICM and the *barangay* health worker when it comes to COVID-19 response” (*PS029_Health Coordinator).*

Overall, the IPC training also built confidence in identifying COVID-19 symptoms so that symptomatic individuals could be referred to BHWs and local health facilities for follow-up and further support.

### Ensuring access to testing for all staff

Access to testing in the Philippines, and globally, has proven challenging during the COVID-19 pandemic. Knowledge of infection status provided through testing is important for community-based actors to safely provide health education, promotion, referrals, and services. To promote access to testing, ICM implemented periodic COVID-19 RT-PCR testing for all staff. Testing was carried out by a private laboratory every three months coinciding with the start of program activities in communities. This pragmatic approach attempted to balance multiple considerations including the limited availability of public testing in the Philippines and the logistical challenges of accessing and processing private test results across areas where ICM operates, while also committing to safe community engagement and working conditions for staff who themselves may have mixed reactions towards testing and concerns around the stigma attached to a positive test.

All Area and Branch Heads reported staff hesitancy to be tested, particularly at the first instance of testing. As one Area Head described, “When we did our swab test not all the staff would want to be tested…The fear of undergoing a swab test. Some would not want to have it, at least they will not know the result” (*PS001_Area Head)*. Reasons for test hesitancy were largely due to fear or hesitation towards completing 14 days of isolation upon testing positive. One Branch Head summed up the emotions of their staff as “Worries especially worries, anxiety [about] what will happen when the result comes. And then before worries, anxieties, what ifs, lots of what ifs” (*PS009_Branch Head).* These worries included concerns about being able to isolate at home given crowded living conditions, as well as concerns around having to stay in government isolation facilities given reports of crowded and unsanitary conditions. Other interviewees reported worrying about infecting others should their test be positive. Some individuals reported concerns about the impact a positive result would have on their family, as close contacts would need to isolate. Many interviewees reflected on the stigma of being tested (regardless of the test outcome). As one Head Trainer reported:

“Because here if they heard that you undergone a swab test, the discrimination is there. Others will think that [if you are tested] you will be positive eventually. And the effect to the family if you are [positive], is that they will be in contact tracing then they will get affected” (*PS003_Head Trainer).*

All Area and Branch Heads reported the importance of communicating early and often with staff to build their confidence in testing. Testing was also an opportunity for leadership to model behaviours to other staff, as the Area Head went onto explain, “I was tested first wherein they could see the courage in me to do it because if you lack courage as a leader, they will not look forward to do it” (*PS001_Area Head).* For some staff, more intensive one-on-one counselling was needed to address their fears and reassure them. As a Branch Head explained, “If ever there are those who are not comfortable, I talked to them personally to let them understand. There are really lots of myths and beliefs about the swab that it’s painful or it will even go to your brain” (*PS012_Branch Head).* Other Branch Heads mentioned how educational trainings, materials, and videos explaining testing helped to address testing hesitancy among staff.

Test processing and results reporting varied across time and geography, with test results reportedly taking between 48 hours and five days. One Area Head described the stress of having positive results communicated by contact tracers from the City Health department, which heightened staff fear. Some leaders described uncertainty about next steps once a positive case was detected and explained that protocols sometimes differed across local government, the DOH, and ICM protocols. As one participant reflected when a staff reported being a close contact of a positive case, “You don’t know what to do because it’s the first time you encounter that. It’s very stressful. I was frustrated…I emailed, I chatted on messenger but I got nothing on what to do, or the advice I needed. (So) we just took initiative [and supported them to quarantine]” (*PS012_Branch Head).*

Staff reflections on testing at ICM underscored the need for testing to be a part of an integrated public health ecosystem linking risk communication, testing, contact tracing and isolation support. Moreover, access to testing needed to be coupled with education targeting the stigma related to COVID-19 and its public health measures in communities, in addition to clearly defined and well-supported next steps for people who test positive and their close contacts.

### Providing support during isolation or quarantine

Isolation of confirmed or suspected cases, and quarantine of close contacts, is key to breaking chains of viral transmission in communities. However, these measures are not without significant financial and social impacts on individuals who must isolate. All participants reflected on the importance of receiving support during quarantine or isolation. Many interviewees reflected on the material and financial support provided by ICM to staff members not only as key to maintaining their livelihoods but also as an example of the sense of community created by the organization. Notably, one Area Head explained how material support only goes so far:

“In case the government cannot provide assistance, we have ICM provided lodging, food and vitamins to strengthen their immune system. Those are all ICM’s assistance to them [who tested positive] but it still hard for their part because of other people’s perception if you tested positive, you are compared to a leper. That’s a very traumatic event” (*PS024_Area Head).*

The emotional effects of the pandemic, and in particular the effects of quarantine or isolation, required additional support [33]. Many participants spoke of how other staff would pray for them and how staff online messaging groups (e.g., WhatsApp chat groups) became a key source of encouragement while in quarantine or isolation. As one participant who tested positive described, “They brought so much food that I can’t consume alone, encouragement, they called me, then they prayed for me” (*PS025_Branch Head).* These relationships were also important in supporting those who had tested positive in returning to work after isolation. As another participant explained:

“When I came back, all of them showed me love. They said that it’s okay, you’re still healthy and you don’t have symptoms. I haven’t felt judgement from them. They really let me feel loved. Everything stayed the same as before. Nothing has changed” (*PS_014 Health trainer).*

Support for quarantine and isolation provided by ICM functioned on two levels: first, to meet the financial and material needs of participants; and second, to provide emotional support and facilitate the creation of positive social bonds. Participants reflected on how both forms of support were key to being able to quarantine or isolate, and thus preventing onward transmission of the virus into the community.

## Discussion

Health systems resilience is ultimately developed in communities. Indeed, at the forefront of fostering resilience are community-based health actors who provide health education, promotion, referrals, and services in deep partnership with those most at need. Drawing on the experiences of individuals employed by one NGO in the Philippines, our study illustrates that while these community-based health actors are crucial in reaching resource-constrained communities, they too must be protected, trained, and supported to safely carry out their roles during public health emergencies. Our analysis reveals that to protect this workforce and foster resilience across the health system, four key areas must be strengthened in organizations providing frontline services: (1) ensuring adequate personal protective equipment (PPE) and hygiene supplies; (2) providing contextualized and role-specific infection prevention and control (IPC) training; (3) ensuring access to testing for all staff; and (4) providing support during quarantine or isolation. These areas, however, are not without their challenges to implementation and maintenance, particularly during a complex and prolonged public health emergency.

Ensuring adequate PPE and hygiene supplies for community-based health actors has been a challenge throughout the COVID-19 pandemic, particularly during early outbreaks and subsequent peaks of infection. Many countries, including the Philippines, reported shortages of PPE, particularly in more rural areas. These shortages arose from increased demand placing unprecedented pressures on global supply chains and manufacturing for these items [34–36]. During emergencies, PPE is prioritized towards hospitals and those providing direct patient care [37]. However, if health workers in communities are to be more fully integrated into the public health response infrastructure, we must consider their PPE needs given the risks of exposure to COVID-19 during community care or health promotion work. Providing adequate PPE to all health workers requires both a strengthened PPE manufacturing supply chain and sufficient stockpiles of PPE accessible to NGOs. If these prove insufficient and PPE is limited in the face of rising case numbers during an outbreak, clear guidance must be provided by national health authorities on PPE repurposing to safely extend the use of existing supplies based on best available evidence. Methods for reuse and extended use are particularly important to ensuring adequate PPE is accessible in resource- constrained settings by all health actors during emergencies. Lessons learned from the COVID-19 pandemic have advanced our understanding of PPE needs during respiratory pandemics as well as offered insights into reusing available resources [38, 39]. Future research should investigate how PPE may be safely and effectively reused at low cost in a variety of settings, including by community-based health actors employed by NGOs providing health and social services in resource-constrained communities.

Protecting community-based health actors is more than the provision of PPE alone. This protection also requires clearly communicated, contextualized, and role-specific IPC training. The provision of such guidelines can prove challenging during emergencies, particularly in fragmented and decentralized health systems such as the Philippines. The novel nature of COVID-19 along with the massive scale up of the health workforce has increased training demands to ensure a skilled and protected workforce. In many countries, including the Philippines, regional or multi-lateral health organizations have provided workforce training, often for workers in the public system or made available through open online platforms such as WHO Open [40–42]. There is little literature describing or evaluating how this guidance has been adapted for community-based settings during emergencies in the Philippines or other LMICs. Understanding the training needs and implementation of such IPC programs will offer important insights towards protecting community-based health actors in these settings [43]. However, as the COVID-19 pandemic has shown, creating this guidance will be challenged by evolving evidence and changing guidance and requires capacity in NGOs to effectively adapt available evidence [28]. Moreover, the creation and implementation of contextualized (including translation) and role-specific IPC training extends to and must be informed by a deep understanding of the socio-political context in which community-based health actors operate. For example, the decentralized health system in the Philippines may contribute to uneven implementation of national guidance and allocation of health resources at the community-level [44]. Consequently, NGOs, in addition to community-based health actors employed by NGOs represent a critical component to health system functioning in some settings through addressing gaps in national and local health capacity. During an extended and uncertain emergency, ongoing communication with community-based health actors about the emerging science and flexible training approaches are key to ensuring training needs are met [45]. Additionally, strengthening linkages between health authorities, researchers, and NGOs before emergencies can create important collaborative channels for assessing needs, developing contextualized guidance, and evaluating its use during emergencies.

Community-based health actors providing health and social care in communities also require access to testing, both for diagnostic and surveillance purposes. Barriers to testing are multifactorial and range from the systems level to the individual. At a systems level, RT-PCR testing has been challenged during the COVID-19 pandemic in the Philippines and elsewhere by cost, insufficient laboratory capacity as a result of workforce limitations, and global shortages of reagents and laboratory supplies [46]. More recently, RT-PCR limitations caused by surging case numbers due to the Omicron variant have also impacted the availability of rapid antigen tests (RATs) for COVID-19. Addressing these limitations again requires multisectoral agreements towards equitable global resource allocation and scale up of manufacturing capacities. Ensuring community-based health actors employed by NGOs can access testing requires agreements between NGOs and health authorities. At the individual level, barriers to testing reported include a fear of an invasive test or positive results. However, our results also highlight a nuanced stigma around testing among staff providing health and social services in communities in the Philippines. While much has been written about the stigma associated with infectious disease outbreaks and an emergent body of literature has formed around stigma associated with testing positive for COVID-19, less is known about stigma associated with the act of COVID-19 testing more generally, regardless of outcome [47, 48]. Tailored and community engaged risk communication strategies that include information on testing may be an important approach towards building confidence in testing and reducing community stigma around those being tested [49].

While quarantine and isolation are foundational to outbreak responses, the COVID-19 pandemic has highlighted the importance of financial, material, and emotional support to ensure people are able to safely adhere to these measures. In settings without robust social safety nets, NGOs may be important providers of support for those quarantining or isolated at home (including community-based health actors employed by NGOs). For those in crowded living conditions, quarantine or isolation facilities have been deployed to prevent household and community transmission and ensure adherence [50, 51]. However, there have been reports of unsafe and unhygienic facilities, as well as unclear discharge procedures for people who have recovered from COVID-19 [52–54]. These reports and experiences during the COVID-19 pandemic, coupled with the carceral history of public health approaches to infectious disease management, compound public hesitancy towards staying at quarantine and isolation facilities [52, 55]. In light of these realities, we argue that it is necessary for various actors (national, municipal, NGOs) to work collaboratively to build local capacity to support quarantine and isolation needs especially in resource- constrained settings where it is not guaranteed that established facilities exist.

Ultimately, overcoming these challenges and realizing the potential of the community-based health workforce towards pandemic responses and health systems resilience demands a commitment to resilience that is not just coping with crisis. Health systems must be transformed to ensure they have the adaptive capacity to respond in communities to complex and intersecting crises. Community-based health actors have an important role in bolstering this adaptive capacity. Yet, to fully realize the potential of this workforce will require a paradigm shift that centres the community in our current conceptualization of who is a part of the health system and where health systems resilience can be fostered [56]. Our findings illustrate the important insights that can be gained when we reimagine and extend our paradigms into communities. We highlight three key insights into health systems resilience gained from this framing. First, health systems resilience is ultimately built on longstanding relationships with communities and the deep contextual knowledge gained from these relationships. Second, resource limitations, particularly during emergencies, hinder the adaptive capacity of the system, particularly at the most local level. Finally, community-based health actors can be better supported to adapt to crises if decision-makers understand their lived realities and policy-to-practice gaps. These lessons are particularly important in countries such as the Philippines and other LMICs where community-based health actors play an important role in delivering health promotion, public health activities, and referral to care to bridge gaps in public care. The future of health systems resilience needs multisectoral action and sustainable global and national investment to developing the health workforce and health service delivery at the local level, by a range of actors, before, during and after public health emergencies.

A strength of our study was the extended timeline for data collection, which allowed us to understand experiences across different stages of the pandemic. Additionally, most interviews were conducted in local languages, leading to more in-depth sharing by participants, particularly around sensitive topics. However, there are several limitations to our study to acknowledge. In particular, the experiences reported reflect operations at one NGO given longstanding relationships between the study team and ICM. Future research would be strengthened by exploring other comparable organizations for similar themes. Additionally, we primarily conducted interviews with staff who were involved in health care adjacent role such as health promotion and referral. Future research could more fully examine how trained health professionals (e.g., nurses, midwives, physicians) employed by NGOs experienced the pandemic and ultimately contribute to health systems resilience.

## Conclusion

Extending health systems resilience in communities is crucial towards ending this pandemic and preventing the next. To foster health systems resilience in communities requires concerted effort and sustained investment in protecting frontline community-based health actors who provide health and social services. To ensure protection, effort and investment should focus on (1) ensuring adequate personal protective equipment (PPE) and hygiene supplies; (2) providing contextualized and role-specific infection prevention and control (IPC) training; (3) ensuring access to testing for all staff; and (4) providing support during quarantine or isolation. Through policy development, training approaches and future research, barriers to health systems resilience in communities can be lowered to ensure equitable delivery of and access to essential health and social services even during complex and prolonged emergencies.

## Supplementary Information

**Additional file 1:** Key informant interview guides.

**Additional file 2:** Summary of interview questions exploring experiences of International Care Ministries’ (ICM) staff during the COVID-19 pandemic.

## Data Availability

The de-identified data (meaning redaction of names, places, and any information deemed potentially identifiable) that support the findings of this study are available on reasonable request from the corresponding author. The data are not publicly available to maintain the confidentiality of research participants given the sensitive nature of the topic area.

## Abbreviations

BHW: barangay health worker
COVID-19: coronavirus disease 2019
DOLE: Department of Labour and Employment
DOH: Department of Health
LMICs: low- and middle-income countries
ICM: International Care Ministries
IPC: infection prevention and control
NGO: non-governmental organization
PPE: personal protective equipment
RT-PCR: reverse transcription polymerase chain reaction
RAT: rapid antigen test
USD: United States dollar
WHO: World Health Organization
