# Supplementary material for: Extending health systems resilience into communities: a qualitative study with community-based actors providing health services during the COVID-19 pandemic in the Philippines
Source: BMC Health Serv Res. 2022 Nov 21;22:1385. doi: 10.1186/s12913-022-08734-4 (PMC9677893; doi:10.1186/s12913-022-08734-4)
Supplement: Supplementary file 1 — Additional file 1: Key informant interview guides. [file 12913_2022_8734_MOESM1_ESM.docx]

**ADDITIONAL FILE 1: KEY INFORMANT INTERVIEW GUIDES**

**Interview Guide for Coordinators & Trainers**

**Introductory questions**

1. Could you start off by telling me a bit about yourself?
2. Can you tell me about your role at ICM?
   1. How long have you been in your current role?
   2. What are your main responsibilities?

**Testing**

1. I understand that ICM is providing COVID-19 testing for staff, can you tell me more details about this?
   1. Where does testing take place? How often are you tested?
2. Why do you think ICM is offering this service?
3. What are your thoughts on ICM offering this service?
4. Do people want to be tested for COVID-19?
   1. Why? Why not?

**Experiences at work**

1. Can you tell me about a typical day at work during the COVID-19 pandemic?
2. How do you prepare to visit communities?
   1. PPE? Training?
3. Do you feel well-prepared to visit communities?
   1. Why? Why not?
4. Can you tell me what activities you do in communities during COVID-19?
5. What is challenging about doing these activities during COVID-19?
   1. Why?
6. I’ve heard that crossing check points is challenging during COVID-19 - can you tell me more about these check points?
   1. What do they ask? What do they check?
   2. How often do you cross check points in your work?
7. While in the communities, what are people’s reactions to health workers?
   1. Do they have a lot of questions? Why?
   2. Are they curious? Why?
   3. Do any have negative reactions? Why?
8. During COVID-19 have you faced any problems during your work as a healthcare worker?
   1. What problems? Why? How often? How do you respond?
9. Have you had any experiences at work during COVID-19 that made you uncomfortable or feel unsafe?
   1. Can you give me an example? Why? How did you respond?
10. Is there anything that could be done to better support you at work during COVID-19?

**Experiences at home**

1. After work, do you take any precautions once you reach home?
   1. Handwashing, changing clothes, showering
   2. Why? Why not?
2. During COVID-19 have you faced any problems in your home or in your community because you are a healthcare worker?
   1. What problems? Why? How often? How do you respond?
3. During COVID-19 have you faced any problems in your home or in your community because you travel to different communities?
   1. What problems? Why? How often? How do you respond?
4. Have you had any experiences in your community during COVID-19 that made you uncomfortable or feel unsafe?
   1. Can you give me an example? Why? How did you respond?
5. Is there anything that could be done to better support you at home or in your community during COVID-19?

**Closing Question**

1. What advice would you give other community health workers during the pandemic?

**Interview Guide for Branch/Area Heads and Admin**

**Introductory questions**

Could you start off by telling me a bit about yourself?

Can you tell me about your role at ICM?

- 1. How long have you been in your current role?
  2. What are your main responsibilities?

**Testing**

1. I understand that ICM is providing COVID-19 testing for staff, can you tell me more details about implementing this service?
   1. Where does testing take place? How often are you and your staff tested?
2. Why do you think ICM is offering this service?
3. What are your thoughts on ICM offering this service?
   1. What has made the service easy to implement?
   2. What has made it hard to implement?
   3. What problems have you faced and how did you solve them?
4. Do people want to be tested for COVID-19?
   1. Why? Why not?

**Experiences at work**

1. Can you tell me about a typical day at work during the COVID-19 pandemic?
2. How have you prepared your staff to visit communities?
   1. PPE? Training?
3. Can you tell me what activities you do in communities during COVID-19?
4. What is challenging about coordinating these activities during COVID-19?
   1. Why?
5. I’ve heard that crossing check points is challenging during COVID-19 - can you tell me more about these check points?
   1. What do they ask? What do they check?
   2. How often do you cross check points in your work?
6. While in the communities, what are people’s reactions to health workers?
   1. Do they have a lot of questions? Why?
   2. Are they curious? Why?
   3. Do any have negative reactions? Why?
7. During COVID-19 have you faced any problems during your work as a coordinator?
   1. What problems? Why? How often? How do you respond?
8. During COVID-19 have your staff reported any problems during their work?
   1. What problems? Why? How often? How do you respond?
9. Have you had any experiences at work during COVID-19 that made you uncomfortable or feel unsafe?
   1. Can you give me an example? Why? How did you respond?
10. Is there anything that could be done to better support you at work during COVID-19?
11. Have your staff reported any experiences at work during COVID-19 where they felt uncomfortable or feel unsafe?
    1. Can you give me an example? Why? How did you respond?
12. Is there anything that could be done to better support your staff at work during COVID-19?

**Experiences at home**

1. After work, do you take any precautions once you reach home?
   1. Handwashing, changing clothes, showering
   2. Why? Why not?
2. During COVID-19 have you faced any problems in your home or in your community because you are a healthcare worker?
   1. What problems? Why? How often? How do you respond?
3. During COVID-19 have you faced any problems in your home or in your community because you travel to different communities?
   1. What problems? Why? How often? How do you respond?
4. Have you had any experiences in your community during COVID-19 that made you uncomfortable or feel unsafe?
   1. Can you give me an example? Why? How did you respond?
5. Is there anything that could be done to better support you at home or in your community during COVID-19?

**Closing Question**

1. What advice would you give others who are coordinating community health work during the pandemic?
