# Supplementary material for: Extending health systems resilience into communities: a qualitative study with community-based actors providing health services during the COVID-19 pandemic in the Philippines
Source: BMC Health Serv Res. 2022 Nov 21;22:1385. doi: 10.1186/s12913-022-08734-4 (PMC9677893; doi:10.1186/s12913-022-08734-4)
Supplement: Supplementary file 2 — Additional file 2: Summary of interview questions exploring experiences of International Care Ministries’ (ICM) staff during the COVID-19 pandemic. [file 12913_2022_8734_MOESM2_ESM.docx]

Box 1. Summary of interview questions exploring experiences of International Care Ministries’ (ICM) staff during the COVID-19 pandemic

| - Could you tell me a bit about your role at International Care Ministries? - So far what has been the impact of COVID-19 on the communities you work in? - What have been people’s reaction to COVID-19 in the communities you work in? - Can you tell me about a typical day at work during the pandemic? - How do you prepare to visit communities/How have you prepared your staff to visit communities? - Can you tell me about the activities in communities during COVID-19? Any adaptations? - Are there any challenges in coordinating/delivering these activities during COVID-19? - Do you have any feedback on the integrated guidelines? - What parts of the guidelines were the most challenging/easiest to implement? - Do the guidelines make you feel more prepared to respond to COVID-19 or other similar outbreaks? - While in communities, what are people’s reactions to health workers? - Is there anything that could be done to better support you at work during COVID-19? - Can you tell me about any programs ICM is providing for staff? - Can you elaborate on details of these services? - What are your thoughts on implementing these services? - What advice would you give others who are working in communities/coordinating community health work during the pandemic? |
| --- |
